# Long-term follow-up of a consecutive cohort validating an epidermal growth factor receptor mutation as an independent risk factor for postoperative recurrence in lung adenocarcinoma

**DOI:** 10.1093/icvts/ivad174

**Published:** 2023-10-31

**Authors:** Yuki Matsumura, Kazuki Hayasaka, Tetsuya Ohira, Satoshi Shiono, Jiro Abe, Hirotsugu Notsuda, Akira Sakurada, Hiroyuki Suzuki, Yoshinori Okada

**Affiliations:** Department of Chest Surgery, Fukushima Medical University, School of Medicine, Fukushima, Japan; Department of Thoracic Surgery, Institute of Development, Ageing and Cancer, Tohoku University, Sendai, Miyagi, Japan; Department of Epidemiology, Fukushima Medical University, School of Medicine, Fukushima, Japan; Department of Thoracic Surgery, Yamagata Prefectural Central Hospital, Yamagata, Japan; Department of Surgery II, Faculty of Medicine, Yamagata University, Yamagata, Japan; Department of Thoracic Surgery, Miyagi Cancer Center, Natori, Miyagi, Japan; Department of Thoracic Surgery, Institute of Development, Ageing and Cancer, Tohoku University, Sendai, Miyagi, Japan; Department of Thoracic Surgery, Institute of Development, Ageing and Cancer, Tohoku University, Sendai, Miyagi, Japan; Department of Chest Surgery, Fukushima Medical University, School of Medicine, Fukushima, Japan; Department of Thoracic Surgery, Institute of Development, Ageing and Cancer, Tohoku University, Sendai, Miyagi, Japan

**Keywords:** Epidermal growth factor receptor mutation, Lung adenocarcinoma, Surgery, Recurrence

## Abstract

**OBJECTIVES:**

Third-generation epidermal growth factor receptor (EGFR) tyrosine kinase inhibitors were recently reported to be effective as adjuvant therapy for resected lung adenocarcinoma (ADC) harbouring common EGFR mutations. However, whether the EGFR mutation is a direct risk factor for postoperative recurrence remains unknown. Therefore, we conducted a multi-institutional observational study to compare postoperative survival according to EGFR mutation status.

**METHODS:**

We collected the medical records of consecutive patients who underwent surgical resection for ADC between 2005 and 2012 at 4 participating institutions. Recurrence-free survival (RFS) and overall survival (OS) associated with EGFR mutation status were evaluated. We further analysed survival after pair-matching patients’ clinicopathological characteristics.

**RESULTS:**

EGFR mutations were harboured by 401 of 840 (48%) enrolled patients. The number of patients with an EGFR mutation (M group) differed from that with the EGFR wild-type sequence (W group) in terms of sex, smoking history and pathological stage. The median follow-up period was 85 months. The five-year RFS/OS rates of the M and W groups were 70%/85% and 61%/75%, respectively (*P* < 0.001 for both groups). However, multivariable analysis revealed that EGFR mutation status was not independently related with both RFS and OS. In pair-matched analysis, the RFS and OS curves of the patients with an EGFR mutation and wild-type sequence were not statistically different, either.

**CONCLUSIONS:**

Long-term follow-up of consecutive patients did not show that a common EGFR mutation was an independent risk factor of recurrence or prognostic factor for completely resected lung ADC.

## INTRODUCTION

An epidermal growth factor receptor (EGFR) mutation is a robust prognostic factor for patients with recurrent or advanced lung adenocarcinoma (ADC) [1]. Several randomized phase III trials have demonstrated that patients harbouring EGFR mutations obtain a survival benefit, mainly because of the beneficial effects of EGFR-tyrosine kinase inhibitors (TKIs) [2, 3]. Moreover, in 2020, the ADAURA trial revealed that third-generation EGFR-TKIs administered as postoperative adjuvant treatment extended recurrence-free survival (RFS) of patients with completely resected EGFR-mutated ADC [4]. However, whether an EGFR mutation directly influences RFS and overall survival (OS) remains unclear. Some investigators claim that an EGFR mutation is a favourable prognostic factor, although others disagree [5, 6]. We also reported that an EGFR mutation is not a risk factor for recurrence in our past analysis [7]. However, these studies had a limitation of a shorter median follow-up period of 49 months, considering that even patients with advanced EGFR-mutated ADCs are reported to have a median OS time of about 40 months nowadays [8]. Additionally, some studies claim that most EGFR-mutated lung ADCs acquire resistance to EGFR-TKIs, while others respond well for a long time [6, 8]. Therefore, we re-collected medical information of the same patients included in our past studies [7, 9] and analysed their survivals. The purpose of the present research was to examine whether an EGFR mutation serves as a risk factor of recurrence or as a prognostic factor of patients with completely resected lung ADC according to our long-term follow-up data.

**Figure:**
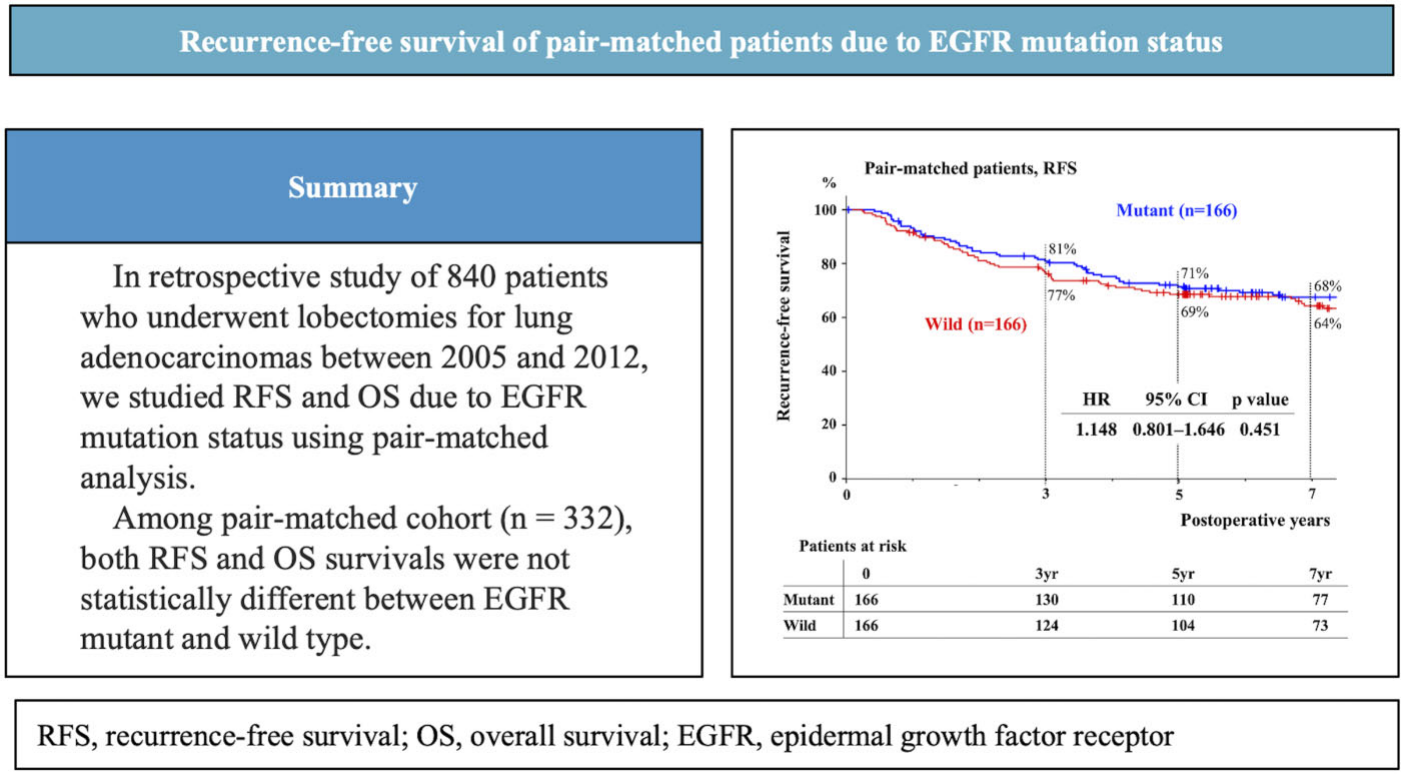


## PATIENTS AND METHODS

### Ethics statement

The current study was newly approved by the institutional review board of the Japanese Northern East Area Thoracic Surgery Study Group and each participating institution. The primary review board was Tohoku University (approval number: 2019-1-188). Formal consent was obtained in the form of opt-out on the website (https://www.med.tohoku.ac.jp/public/doc/old2019/).

### Study design and patient enrolment

The current study was an additional analysis of our past reports [7, 9]. Details of the study design, including the sample size required for the analysis, were also described in them. According to the inclusion and exclusion criteria, the medical information of consecutive patients who underwent complete resections of lung ADC between January 2005 and December 2012 was collected from the 4 participating institutions (Supplementary Material, Fig. S1). In this study, complete resection was defined as radical tumour resection without macroscopic or microscopic remnants. All patients’ tumours were examined for EGFR mutations. The primary end point was the RFS of patients with EGFR mutation (M group) and those with wild type (W group). The secondary end point was their OS. Pathological diagnosis was determined by the TNM classification of malignant tumours of the Union for International Cancer Control (7th Edition) [10] and the World Health Organization classification (4th Edition) [11].

The medical records of 1047 enrolled patients were collected. We excluded patients who underwent limited resection, that is, wedge resection or segmentectomy because, as of 2012, there was no established evidence of limited resection as curative surgery. The patients harbouring uncommon EGFR mutations, as defined below, were excluded because their clinical behaviours are heterogenous, which was inconsistent with our aim of determining the biological features of EGFR-mutated lung ADC [12]. Because an EGFR mutation is the most common driver gene mutation, the statuses of other targetable gene mutations (e.g. *KRAS, ALK, ROS1, RET* and *BRAF*) were not required to be recorded. Finally, we enrolled 840 patients in the current analysis.

### Analysis and evaluation of EGFR mutations

Each institution utilized an individual commercial method to examine EGFR mutations of the tumours. Regarding the inter-institutional difference of method for determining EGFR mutations, as we described in our past report, the difference would have few effects on the rate of EGFR mutation status detection because these methods are generally performed in the clinic and their performances were reported comparable [7, 13]. In the present study, the exon 21 L858R and exon 19 deletion were defined as common EGFR mutations, and we excluded other uncommon mutations such as exon 18 G719A, *de novo* exon 20 T790M and exon 21 L861Q (Supplementary Material, Table S1). The patients with dual EGFR mutations were also excluded from the current analysis.

### Follow-up and evaluation of recurrence

Postoperative adjuvant therapy and therapy for recurrence followed the Japanese guidelines for the diagnosis and treatment of lung cancer, which is annually published by the Japan Lung Cancer Society [14]. Oral chemotherapy in Table 1 represents tegafur (UFT; Taiho, Tokyo, Japan) for pathological stage (pStage) IB, which is recommended by the guidelines and evidence-based reports [15]. We excluded the patients who were treated by EGFR-TKIs before recurrence from the current analysis, despite they were administered pre- or postoperatively. Without recurrence, the patients were usually followed in the outpatient clinic every 3–6 months for 5 years after surgery. Each visit included a physical examination, blood test with tumour markers and chest X-ray. Chest computed tomography scans were performed once or twice a year for examining recurrence. Thereafter, the visit interval was extended to once a year. Postoperative recurrence, including the differential diagnosis of pulmonary metastases and multiple lung cancers, was determined by a multidisciplinary tumour board at each institution according to the findings of physical examination, chest and abdominal computed tomography, brain magnetic resonance imaging or positron emission tomography. The date of recurrence was defined as the date of histological diagnosis or the date of diagnosis according to clinicoradiological findings. Histological confirmation of recurrence was not required.

**Table 1:** Clinicopathological characteristics and therapeutic outcomes of all enrolled patients (*n* = 840)

Characteristic		EGFR mutant (M), *n* = 401 (%)	EGFR wild type (W), *n* = 439 (%)	*P*-value	Std diff
Age (years)	Median (IQR)	67 (61–74)	68 (61–75)	0.50	0.005
Sex	Men	131 (33)	278 (63)	<0.001	0.63
Smoking history	Ever smoker	112 (28)	283 (65)	<0.001	0.80
Smoking index	Median PY (IQR)	0 (0–5)	22 (0–44)	<0.001	0.027
	PY 20 or more	43 (11)	146 (33)	<0.001	0.55
Serum CEA	>5 ng/ml	72 (18)	118 (27)	0.002	0.22
Pathological stage	IA	237 (59)	190 (43)	0.001	0.23
	IB	83 (21)	117 (27)	I versus II more	
	II	42 (10)	75 (17)		
	III	39 (10)	57 (13)		
WHO classification	AIS	22 (6)	25 (6)	0.070	0.085
	MIA	41 (10)	32 (7)	IA versus others	
	IA^a^	336 (84)	382 (87)		
Pleural inv.	Present	72 (18)	115 (26)	0.005	0.19
Vascular inv.	Present	67 (17)	98 (22)	0.046	0.13
Lymphatic per.	Present	85 (21)	101 (23)	0.56	0.048
Adjuvant	None	296 (74)	296 (67)	0.039	0.15
Treatment	Oral	55 (14)	76 (17)	Adj versus non-adj	
	Platinum doublet	48 (12)	62 (14)		
	Others	2 (1)	5 (1)		
Recurrence	Total	118 (29)	167 (38)		
	Intrathoracic	59 (15)	93 (21)	0.29	0.028
	Extrathoracic	59 (15)	72 (16)	Intra versus extra	
	Unknown	0	2 (1)		
Postrec TKI	Administered	96 (81)^a^	20 (12)^b^	<0.001	1.916
EGFR mutation	Exon 21 L858R	225 (56)	NA		
	Exon 19 del	174 (43)	NA		
	Both 21 and 19	2 (1)	NA		

a Of 118 recurrent patients.

b Of 167 recurrent patients.

AIS: adenocarcinoma in situ; CEA: carcinoembryonic antigen; EGFR: epidermal growth factor receptor; IA: invasive adenocarcinoma; inv.: invasion; IQR: interquartile range; MIA: minimally invasive adenocarcinoma; NA: not available; per.: permeation; postrec TKI: post-recurrence tyrosine kinase inhibitors administered; PY: pack-year; Std diff: standardized difference; WHO: World Health Organization.

### Statistical analysis

Pair-matching analysis was adopted to mitigate clinicopathological bias between EGFR mutant and wild-type sequences, considering our past findings [7]. For each patient in the M group, 1 patient in the W group was randomly selected without repetition. Then, with prioritization of hard-to-match factors, patients were matched according to institution, gender, age (±3 years), smoking history (never versus ever smoker), pStage (IA versus IB versus II versus III) and adjuvant treatment (absent versus present). The adjuvant treatment ‘present’ group represented the patients who were administered any kind of chemotherapy postoperatively for preventing recurrence. For age, a range of ±3 years was established because it represented one-fourth of the standard deviation. Other matching factors were established using exact matching. Patient matching was conducted using SAS version 9.1 (SAS Institute, Cary, NC, USA) by one of the authors (Tetsuya Ohira), who only had information regarding the matching factors described above. Selection yielded 166 patients each with mutant or wild-type EGFR.

Differences between groups were evaluated by Fisher’s exact test or Pearson’s χ^2^ test. We defined RFS as the interval in months between the date of resection and the date of first recurrence or last follow-up. Non-cancer death was also regarded as an event of RFS. OS was defined as the interval in months between the date of resection and the date of death from any cause or the last follow-up. Among the recurrent patients, we defined post-recurrence survival (PRS) as the interval in months between the date of recurrence and the date of death from any cause or the last follow-up. Observations were censored at the last follow-up when the patient was alive or lost to follow-up.

RFS, OS and PRS were plotted with the Kaplan–Meier method, and statistical differences between 2 groups were analysed using the log-rank test. We selected risk factors and prognostic factors for survival analysis from published literature [16–18]. Statistically significant factors identified during univariable analysis were included in multivariable analysis using Cox proportional hazard regression models. Its goodness of fit was evaluated by Harrell’s *C*-index, and the variance inflation factor was utilized to assess multicollinearity. All *P*-values were two-sided, and *P *<* *0.05 was defined as a statistically significant difference. Statistical analysis was performed using SPSS statistical software (IBM Corp, Released 2020, IBM SPSS Statistics for Windows, Version 27.0; IBM Corp., Armonk, NY).

## RESULTS

### Patients’ clinicopathological characteristics and survival

Clinicopathological backgrounds of the enrolled patients (*n* = 840) are shown in Table 1 and Supplementary Material, Tables S2 and S3. The M group comprised 401 (48%) patients and included fewer men, fewer smokers and more with pStage I, compared with the W group. The median follow-up time among all patients was 85 months (interquartile range: 55–115 months), in which 285 (34%) patients recurred (Table 1) and 250 (30%) patients died. The last update of OS was 30 April 2021; 448 (76%) of the 590 surviving patients had been followed up for 5 years or more. There was no statistical difference in the numbers of intra- and extrathoracic recurrences between the M and W groups (*P *=* *0.29), while the M group was administered EGFR-TKIs more frequently than the W group.

**Figure 1:**
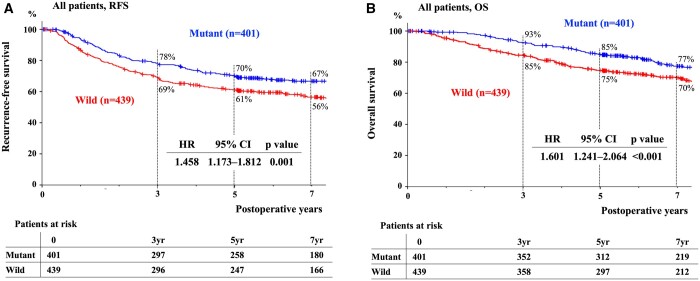
RFS and OS of all enrolled patients according to EGFR mutations. The 5-year RFS (**A**)/OS (**B**) rates of patients harbouring an EGFR mutant or wild-type sequences were 70%/85% and 61%/75%, respectively. EGFR: epidermal growth factor receptor; OS: overall survival; RFS: recurrence-free survival.

**Figure 2:**
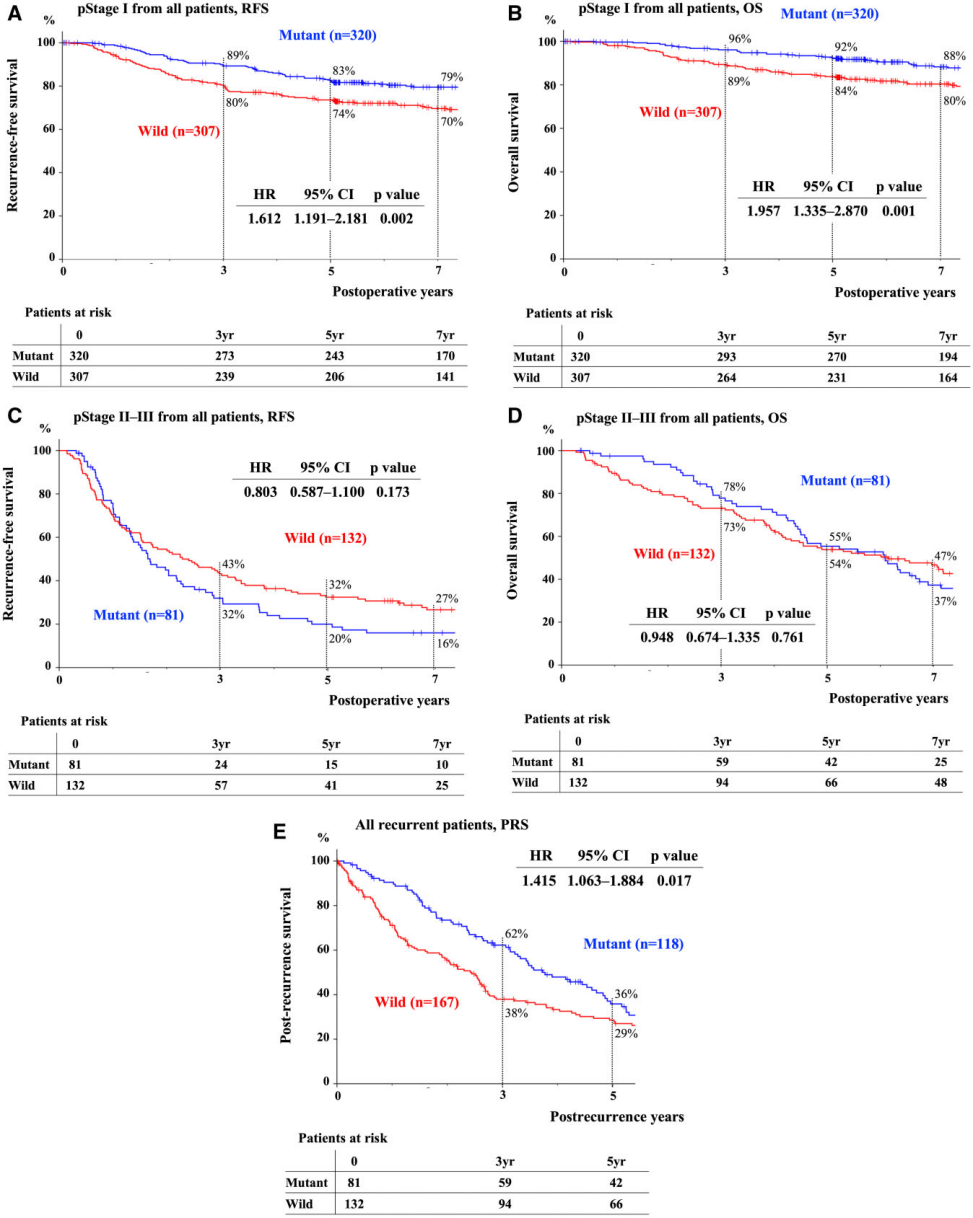
RFS, OS and PRS of all patients according to EGFR mutations and pStage. In pStage I from all patients, the 5-year RFS (**A**)/OS (**B**) rates of patients with an EGFR mutation or wild type were 83%/92% and 74%/84%, respectively. In pStage II–III, the 5-year RFS (**C**)/OS (**D**) rates of patients with an EGFR mutation or wild type were 20%/55% and 32%/54%, respectively. (**E**) The 3-year PRS rates of patients with an EGFR mutation or wild type were 62% and 38%, respectively. EGFR: epidermal growth factor receptor; OS: overall survival; PRS: post-recurrence survival; pStage: pathological stage; RFS: recurrence-free survival.

In Fig. 1, the RFS and OS curves according to EGFR mutation status are shown. The 5-year RFS/OS rates of the M and W groups were 70%/85% and 61%/75%, respectively, and both survival curves statistically differed (*P = *0.001). Restricted to pStage I, the 5-year RFS/OS rates of the M and W groups were 83%/92% and 74%/84%, respectively (RFS; *P *=* *0.002, OS; *P *<* *0.001) (Fig. 2A and B). The 5-year RFS/OS rates of the M and W groups with pStage II–III were 20%/55% and 32%/54%, respectively (RFS; *P *=* *0.17, OS; *P *=* *0.76) (Fig. 2C and D). The 3-year PRS rates of the M and W groups were 62% and 38%, respectively, which were statistically different (*P = *0.017) (Fig. 2E).

The results of univariable and multivariable analyses of RFS, OS and PRS are shown in Table 2. We confirmed the proportional risk of each factor according to the shapes of its RFS, OS and PRS curves (Supplementary Material, Fig. S2). Harrell’s *C*-index values for RFS, OS and PRS were 0.750, 0.761 and 0.653, respectively. All variance inflation factor values were <3 (Supplementary Material, Table S4). Independent risk factors of recurrence were serum carcinoembryonic antigen, pStage, vascular invasion and lymphatic permeation. Independent prognostic factors were age, sex, pStage and vascular invasion. Independent post-recurrence prognostic factors were age, gender and vascular invasion. An EGFR mutation was neither an independent risk factor nor a prognostic factor in all multivariable analyses. The use of EGFR-TKIs was not also an independent factor in multivariable analyses of OS or PRS (Supplementary Material, Table S5).

**Table 2:** Univariable and multivariable analyses of clinicopathological factors associated with recurrence-free, overall survival and post-recurrence survival

Variable	Favourable	Unfavourable	Univariable analysis			Multivariable analysis		
			HR	95% CI	*P*-Value	HR	95% CI	*P*-Value
(A) Recurrence-free survival								
Age	<70	70 more	1.231	0.993–1.527	0.058			
Gender	Woman	Man	1.559	1.256–1.934	<0.001	1.064	0.772–1.466	0.70
Smoking	Never	Ever	1.551	1.251–1.923	<0.001	1.092	0.771–1.544	0.62
Heavy smoker	<20 PY	≥20 PY	1.860	1.477–2.341	<0.001	1.230	0.907–1.670	0.18
CEA	5 less	>5	2.104	1.671–2.650	<0.001	1.402	1.089–1.805	0.009
pStage	I	II or III	5.005	4.030–6.217	<0.001	3.334	2.583–4.303	<0.001
Pleural inv.	Absent	Present	2.408	1.919–3.023	<0.001	1.263	0.982–1.624	0.069
Vascular inv.	Absent	Present	2.781	2.203–3.510	<0.001	1.331	1.016–1.743	0.038
Lymphatic per.	Absent	Present	3.175	2.541–3.966	<0.001	1.681	1.289–2.191	<0.001
Adjuvant therapy	None	Received	2.162	1.741–2.686	<0.001	1.045	0.820–1.332	0.72
EGFR mutation	Mutant	Wild type	1.458	1.173–1.812	0.001	1.025	0.808–1.301	0.84
(B) Overall survival								
Age	<70	70 more	1.595	1.243–2.046	<0.001	1.501	1.152–1.954	0.003
Gender	Woman	Man	2.020	1.564–2.608	<0.001	1.498	1.051–2.135	0.026
Smoking	Never	Ever	1.825	1.418–2.384	<0.001	0.997	0.673–1.477	0.99
Heavy smoker	<20 PY	≥20 PY	2.134	1.643–2.771	<0.001	1.362	0.963–1.926	0.080
CEA	5 less	>5	1.928	1.473–2.523	<0.001	1.046	0.775–1.412	0.77
pStage	I	II or III	4.823	3.755–6.194	<0.001	3.588	2.662–4.836	<0.001
Pleural inv.	Absent	Present	2.216	1.703–2.884	<0.001	1.290	0.970–1.717	0.080
Vascular inv.	Absent	Present	3.061	2.355–3.978	<0.001	1.605	1.184–2.176	0.002
Lymphatic per.	Absent	Present	2.551	1.970–3.302	<0.001	1.212	0.893–1.645	0.22
Adjuvant therapy	None	Received	1.746	1.356–2.247	<0.001	0.891	0.666–1.192	0.44
EGFR mutation	Mutant	Wild type	1.601	1.241–2.064	<0.001	1.092	0.827–1.442	0.54
(C) Post-recurrence survival								
Age	<70	70 more	1.768	1.332–2.346	<0.001	1.708	1.284–2.271	<0.001
Gender	Woman	man	1.988	1.493–2.647	<0.001	1.676	1.147–2.451	0.008
Smoking	Never	ever	1.730	1.303–2.296	<0.001	1.105	0.720–1.695	0.65
Heavy smoker	<20 PY	≥20 PY	1.675	1.244–2.254	0.001	1.085	0.732–1.609	0.68
CEA	5 less	>5	1.135	0.841–1.531	0.41			
pStage	I	II or III	1.367	1.026–1.820	0.032	1.176	0.867–1.594	0.30
Pleural inv.	Absent	Present	1.233	0.924–1.646	0.16			
Vascular inv.	Absent	Present	1.542	1.154–2.061	0.003	1.465	1.076–1.995	0.015
Lymphatic per.	Absent	Present	1.006	0.758–1.336	0.96			
Adjuvant therapy	None	Received	0.822	0.621–1.088	0.17			
EGFR mutation	Mutant	Wild type	1.415	1.063–1.884	0.017	1.158	0.845–1.587	0.36

CEA: carcinoembryonic antigen; CI, confidence interval; EGFR: epidermal growth factor receptor; HR, hazard ratio; inv., invasion; per., permeation; pStage: pathological stage; PY, pack-year.

### Clinicopathological characteristics and survival of pair-matched patients

**Figure 3:**
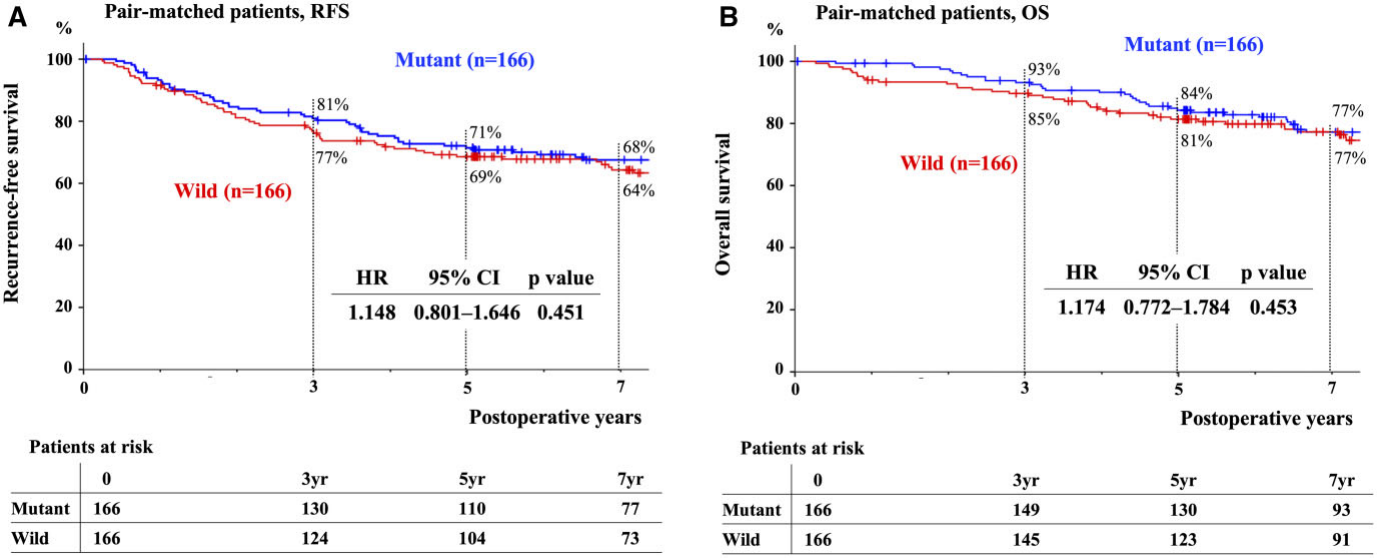
RFS and OS of pair-matched patients according to EGFR mutations. Among the pair-matched cohort, the 5-year RFS (**A**)/OS (**B**) rates of patients with an EGFR mutation or wild type were 71%/84% and 69%/81%, respectively. EGFR: epidermal growth factor receptor; OS: overall survival; RFS: recurrence-free survival.

**Figure 4:**
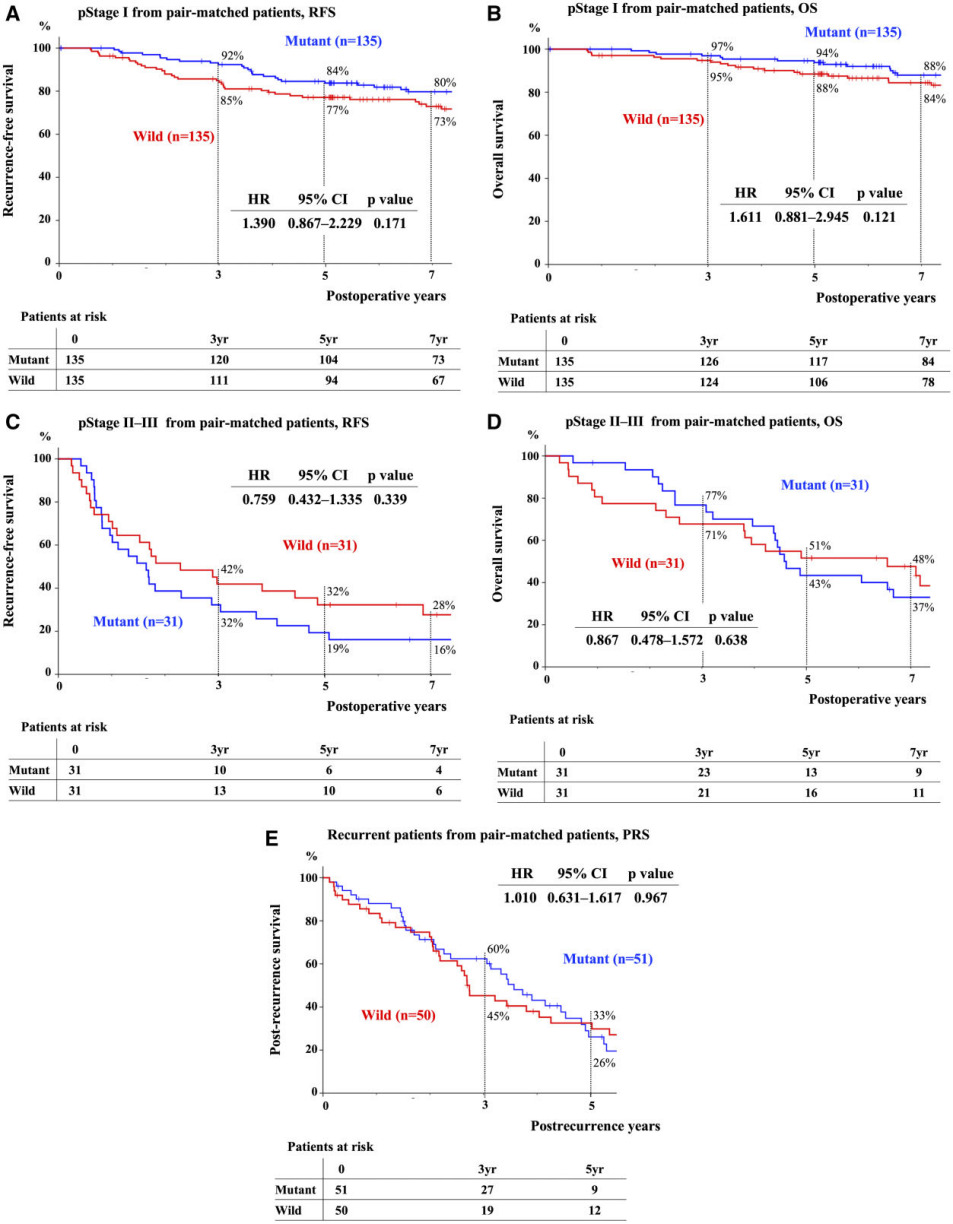
RFS, OS and PRS of pair-matched patients according to EGFR mutations and pStage. In pStage I from pair-matched patients, the 5-year RFS (**A**)/OS (**B**) rates of patients with an EGFR mutation or wild type were 84%/94% and 77%/88%, respectively. In pStage II–III, the 5-year RFS (**C**)/OS (**D**) rates of patients with an EGFR mutation or wild type were 19%/43% and 32%/51%, respectively. (**E**) The 3-year PRS rates of patients with an EGFR mutation or wild type were 60% and 45%, respectively. EGFR: epidermal growth factor receptor; OS: overall survival; PRS: post-recurrence survival; pStage: pathological stage; RFS: recurrence-free survival.

As described in our past report, the patients were assigned into 2 groups (*n* = 166 each) with the EGFR mutant (mM group) or wild-type (mW group) sequence according to the matching institution, sex, smoking history, pStage and adjuvant treatment (Supplementary Material, Table S6) [7]. All clinicopathological factors were comparable between both groups, except recurrence pattern and post-recurrence TKI administered. In the matched groups, 101 (30%) patients had recurrence during the follow-up period. The RFS and OS curves of the pair-matched patients are presented in Fig. 3. The 5-year RFS/OS rates of the mM and mW groups were 71%/84% and 69%/81%, respectively (RFS, *P = *0.45; OS, *P = *0.45) (Fig. 3A and B). In a subgroup of pStage I of the pair-matched cohort, the 5-year RFS/OS rates of the mM and mW groups were 84%/94% and 77%/88%, respectively (RFS; *P *=* *0.17, OS; *P = *0.12) (Fig. 4A and B). Among the pair-matched patients with pStage II–III, the 5-year RFS/OS rates of the mM and mW groups were 19%/43% and 32%/51%, respectively (RFS; *P *=* *0.34, OS; *P *=* *0.64) (Fig. 4C and D). The 3-year PRS rates of the mM and mW groups were 60% and 45%, respectively (*P = *0.97) (Fig. 4E).

## DISCUSSION

An EGFR mutation is a documented prognostic factor for patients with recurrent or advanced lung ADCs [1], and several randomized phase III trials demonstrate that patients with EGFR-mutated lung ADCs gain longer survival than those with wild type through the effects of EGFR-TKIs [2, 3]. Furthermore, the ADAURA trial proposed the efficacy of the third-generation EGFR-TKI, or osimertinib as postoperative adjuvant chemotherapy for resected lung ADC harbouring EGFR mutations [4]. However, it still remains unclear whether an EGFR mutation is a direct risk factor of recurrence after complete resection for lung ADCs [16, 19]. Several studies propose that an EGFR mutation is a favourable factor for postoperative recurrence [6, 16], while others propose that postoperative survival of lung ADCs is not substantially different independent of EGFR mutation status [6]. Here, we used long-term follow-up data to answer this primary clinical question.

The most important finding was that an EGFR mutation was neither independently related with postoperative recurrence nor prognosis. Univariable analysis showed that RFS and OS of the M group were statistically better than those of the W group, although multivariable analyses of RFS and OS did not reveal that an EGFR mutation was an independent favourable risk factor for recurrence or prognostic factor. Those results are consistent with some other reports suggesting that the RFS rates of EGFR-mutated and EGFR-wild-type ADC were comparable [16, 19]. An EGFR mutation is the most frequent driver of genetic variant in Asian patients with ADCs, while tumours with the EGFR wild-type sequence probably harboured other driver gene alterations such as *KRAS, ALK, ROS1, RET*, or *BRAF*, and their biological behaviour is considered similar to that of EGFR-mutated ADCs [20, 21].

Additionally, in the pair-matched patients, both RFS and OS curves between the mM group and the mW group were comparable. In this cohort, PRS of the mM group was not also statistically different, compared with that of the mW group. These results were striking to us because the OS of patients with the EGFR mutation had been anticipated to be better than that of wild-type sequence, considering the beneficial effects of EGFR-TKIs on patients with EGFR-mutated ADC [2, 3, 5]. One possible rationale is that EGFR-TKIs are unable to suppress advanced or recurrent lung ADCs for long time [22]. Since the median progression-free survival time of EGFR-TKIs for those patients is reported to be 10–19 months [3, 23], long-term follow-up data like the current study are possibly affected little by EGFR-TKIs. Differences in the recurrence pattern may have also influenced the OS of both groups. Some investigators proposed that patients with lung ADC who harbour EGFR mutations were at risk of distant metastasis [9, 24], and in our cohort, there were also statistically more extrathoracic recurrences in the mM group compared with the mW group (Supplementary Material, Table S6), which may impair the OS of the mM group [9, 25].

Restricted to pStage I, in the current analysis, the patients with EGFR mutation showed better RFS curve than those with wild type, while in pStage II–III, the patients with EGFR mutation tended to have impaired RFS compared with those with wild type. The difference of RFS due to pStage has been discussed in several past papers. Yotsukura *et al.* [26] reported a better RFS of the patients with EGFR mutation than those with wild type in pStage I–II ADC, while Takamochi *et al.* [27] revealed that multivariable analysis for RFS identified that EGFR mutation status was not an independent risk factor for recurrence. Deng *et al.* [28] proposed that EGFR mutation was a poor prognostic factor in RFS for the patients with pStage II–III. It remains unclear why such differences in RFS due to pStage were observed; however, 1 possible reason is that more ground grass nodules might be observed in EGFR-mutated patients with pStage I because they were reported to have a positive relationship with EGFR-mutated ADC [29] and show favourable RFS [16].

As for the study design of the current study, we predominantly focused on EGFR mutations without identifying other oncogenic driver mutations or fusion genes such as *ALK, BRAF, ROS1, RET* or *KRAS* [20, 21], because the study's retrospective and observational nature did not allow us to examine those gene alterations. However, the EGFR mutation is the most common driver gene mutation harboured by Asian patients with ADC, which occurs at frequencies ranging from 50% to 60% [30]. Therefore, we considered it reasonable to focus on EGFR mutations to better understand the association between driver mutations and prognosis of ADC. The reason we utilized pair-matched analysis to mitigate the bias of patients’ background was already narrated in our past paper [7]. Here, we only underline that pair-matching analysis enabled us to compare survival after matching several clinicopathological factors between 2 groups.

The strong point of the present analysis is that the patients were enrolled consecutively, all of whose tumours were analysed for EGFR mutations unless they were judged insufficient for detecting EGFR mutations. The median follow-up period of 85 months is considered sufficient for survival analysis. The limitations include its retrospective nature and usage of the data collected from different institutions, which did not enable us to standardize follow-up interval, adjuvant treatment, diagnosis of recurrence and post-recurrence therapy. The lack of information about the extent of lymph node dissection might have affected accurate staging in the current research. Histological proof of recurrence was not mandatory, and the pathological findings did not go through a centralized review. Therefore, we did not collect pathological information about predominant subtypes in some patients, and some recurrences may represent metachronous multiple lung cancers. To mitigate such potential sources of bias to the extent possible, we included the institution as one of the matching variables.

## CONCLUSION

Our long-term follow-up data of consecutive patients from multiple institutions and pair-matched analysis did not provide convincing evidence that the presence of a common EGFR mutation is an independent risk factor for the recurrence of completely resected ADC.

## Supplementary Material

ivad174_Supplementary_Data

## Data Availability

The data that support the findings of this study are available from the corresponding author (Yuki Matsumura), upon reasonable request.

## Notes

Presented at the 2022 World Conference of Lung Cancer, Vienna, Austria.

## ABBREVIATIONS

ADC: Adenocarcinoma
EGFR: Epidermal growth factor receptor
OS: Overall survival
PRS: Post-recurrence survival
pStage: Pathological stage
RFS: Recurrence-free survival
TKIs: Tyrosine kinase inhibitors
