# Supplementary material for: Long-term follow-up of a consecutive cohort validating an epidermal growth factor receptor mutation as an independent risk factor for postoperative recurrence in lung adenocarcinoma
Source: Interdiscip Cardiovasc Thorac Surg. 2023 Oct 31;37(5):ivad174. doi: 10.1093/icvts/ivad174 (PMC10640389; doi:10.1093/icvts/ivad174)
Supplement: ivad174_Supplementary_Data [file ivad174_supplementary_data.zip › EGFR long_match_supl table_EJCTSR0_20221210.docx]

**Supplemental table 1. Clinicopathological characteristics of patients (n = 285) with recurrent disease**

| Characteristic |  | EGFR | EGFR | *p* value |
| --- | --- | --- | --- | --- |
|  |  | mutant | wild-type |  |
|  |  | n = 118 (%) | n = 167 (%) |  |
| Age | Median yr (range) | 66 (37–82) | 69 (45–88) | 0.086 |
| Gender | Men | 80 (68) | 52 (31) | <0.001 |
| Smoking history | Ever smoker | 33 (28) | 117 (70) | <0.001 |
| Smoking index | mean PY (range) | 12 (0–200) | 30 (0–129) | <0.001 |
|  | PY 20 or more | 16 (14) | 70 (42) | <0.001 |
| Serum CEA | >5 ng/ml | 34 (29) | 59 (36) | 0.250 |
| Pathological | IA | 27 (23) | 36 (22) | 1.000 |
| stage | IB | 30 (25) | 44 (26) | I vs II more |
|  | II | 10 (27) | 47 (28) |  |
|  | III | 34 (29) | 40 (24) |  |
| WHO | AIS | 0 | 1 (1) | 0.331 |
| classification | MIA | 1 (1) | 3 (2) | IA vs others |
|  | IA* | 116 (98) | 163 (98) |  |
| Pleural inv. | Present | 40 (34) | 63 (38) | 0.533 |
| Vascular inv. | Present | 40 (34) | 51 (30) | 0.606 |
| Lymphatic per. | Present | 50 (42) | 66 (39) | 0.714 |
| Adjuvant | None | 57 (48) | 95 (57) | 0.185 |
| treatment | Oral | 21 (18) | 30 (18) | Adjuvant vs |
|  | Platinum | 40 (34) | 42 (25) | Non-adjuvant |
|  | Others | 2 (2) | 1 (1) |  |
| Interval | median mo (range) | 23 (4–136) | 19 (2–129) | 0.231 |
| Rec pattern | Intrathoracic | 59 (50) | 93 (56) | 0.289 |
|  | Extrathoracic | 59 (59) | 72 (43) | Intra vs Extra |
|  | Unknown | 0 | 2 (1) |  |
| Postrec TKI | Administered | 96 (81) | 20 (12) | <0.001 |
| EGFR mutation | Exon 21 L858R | 57 (48) | NA |  |
|  | Exon 19 del | 59 (50) | NA |  |
|  | Both exon 19 &21 | 2 (2) | NA |  |

PY, pack-year; CEA, carcinoembryonic antigen; AIS, adenocarcinoma in situ; MIA, minimally invasive adenocarcinoma; IA*, invasive adenocarcinoma; inv, invasion; per, permeation; postrec TKI, post-recurrence tyrosine kinase inhibitors administered; NA, not available

**Supplemental table 2. Clinicopathological characteristics of pair-matched patients (n = 332)**

| Characteristic |  | EGFR mut | EGFR wild | *p* value |
| --- | --- | --- | --- | --- |
|  |  | matched (mM) | matched (mW) |  |
|  |  | n = 166 (%) | n = 166 (%) |  |
| Institution | A | 23 (14) | 23 (14) | 1.000 |
|  | B | 62 (37) | 62 (37) |  |
|  | C | 48 (29) | 48 (29) |  |
|  | D | 33 (20) | 33 (20) |  |
| Age | Median yr (range) | 68 (43–84) | 68 (43–83) | 0.962 |
| Gender | Men | 72 (43) | 72 (43) | 1.000 |
| Smoking history | Ever smoker | 65 (39) | 65 (39) | 1.000 |
| Smoking index | mean PY (range) | 14 (0–200) | 15 (0–111) | 0.730 |
|  | PY 20 or more | 25 (15) | 27 (16) | 0.880 |
| Serum CEA | >5 ng/ml | 29 (18) | 38 (23) | 0.274 |
| Pathological | IA | 91 (55) | 91 (55) | 1.000 |
| stage | IB | 44 (26) | 44 (26) | I vs II more |
|  | II | 16 (10) | 16 (10) |  |
|  | III | 15 (9) | 15 (9) |  |
| WHO | AIS | 8 (5) | 15 (9) | 0.169 |
| classification | MIA | 11 (7) | 13 (8) | IA vs others |
|  | IA* | 145 (87) | 138 (83) |  |
| Pleural inv. | Present | 31 (19) | 34 (20) | 0.782 |
| Vascular inv. | Present | 22 (13) | 27 (16) | 0.536 |
| Lymphatic per. | Present | 29 (17) | 29 (17) | 1.000 |
| Adjuvant | None | 123 (74) | 123 (74) | 1.000 |
| treatment | Oral | 29 (17) | 27 (16) | Adjuvant vs |
|  | Platinum | 14 (8) | 16 (9) | Non-adjuvant |
|  | Non-platinum | 0 (0) | 1 (1) |  |
| Recurrence | Total | 51 (31) | 50 (30) | 1.000 |
|  | Intrathoracic | 23 (14) | 34 (20) | 0.020 |
|  | Extrathoracic | 28 (17) | 16 (10) | Intra vs Extra |
| Postrec TKI | Administered | 38 (74)* | 8 (16)** | <0.001 |
| EGFR mutation | Exon 21 L858R | 88 (53) | NA |  |
|  | Exon 19 del | 76 (46) | NA |  |
|  | Both exon 19 &21 | 2 (1) | NA |  |

PY, pack-year; CEA, caracinoembryonic antigen; AIS, adenocarcinoma in situ; MIA, minimally invasive adenocarcinoma; IA*, invasive adenocarcinoma; inv, invasion; per, permeation; post-recurrence TKI, post-recurrence TKI; NA, not available, *of 51 recurrent patients, **of 50 recurrent patients
