# Supplementary material for: Long-term follow-up of a consecutive cohort validating an epidermal growth factor receptor mutation as an independent risk factor for postoperative recurrence in lung adenocarcinoma
Source: Interdiscip Cardiovasc Thorac Surg. 2023 Oct 31;37(5):ivad174. doi: 10.1093/icvts/ivad174 (PMC10640389; doi:10.1093/icvts/ivad174)
Supplement: ivad174_Supplementary_Data [file ivad174_supplementary_data.zip › Suppl fig1.pdf]

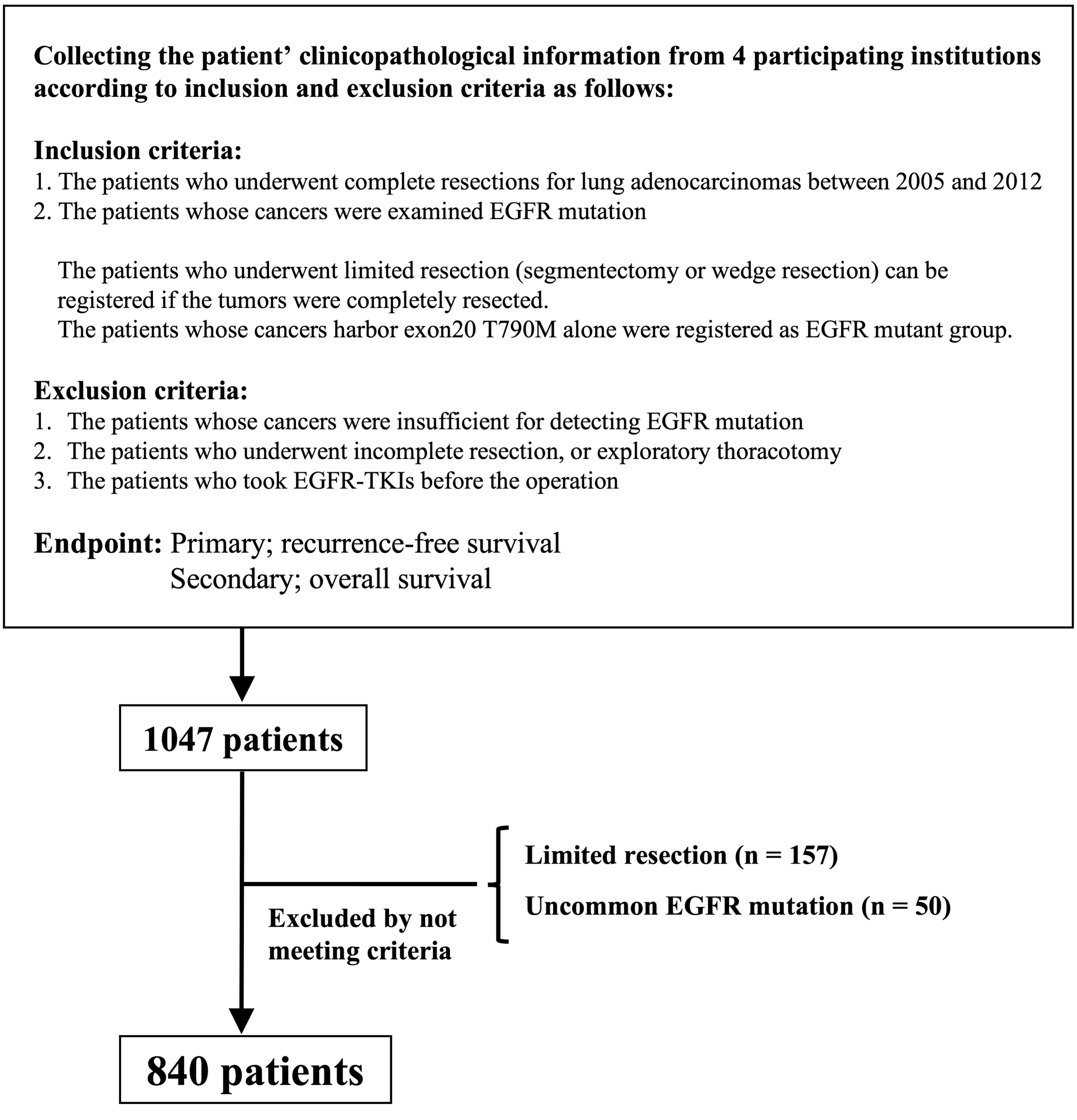
Collecting the patient' clinicopathological information from 4 participating institutions according to inclusion and exclusion criteria as follows:
Inclusion criteria:
1. The patients who underwent complete resections for lung adenocarcinomas between 2005 and 2012
2. The patients whose cancers were examined EGFR mutation
The patients who underwent limited resection (segmentectomy or wedge resection) can be registered if the tumors were completely resected.
The patients whose cancers harbor exon20 T790M alone were registered as EGFR mutant group.
Exclusion criteria:
1. The patients whose cancers were insufficient for detecting EGFR mutation
2. The patients who underwent incomplete resection, or exploratory thoracotomy
3. The patients who took EGFR-TKIs before the operation
Endpoint: Primary; recurrence-free survival
Secondary; overall survival
1047 patients
Limited resection (n = 157)
Uncommon EGFR mutation (n = 50)
Excluded by not meeting criteria
840 patients
