# Supplementary material for: Long-term follow-up of a consecutive cohort validating an epidermal growth factor receptor mutation as an independent risk factor for postoperative recurrence in lung adenocarcinoma
Source: Interdiscip Cardiovasc Thorac Surg. 2023 Oct 31;37(5):ivad174. doi: 10.1093/icvts/ivad174 (PMC10640389; doi:10.1093/icvts/ivad174)
Supplement: ivad174_Supplementary_Data [file ivad174_supplementary_data.zip › EGFR long_match_leg supl fig_ICVTS_R1_2_CL_20230927.docx]

**Figure legends of supplemental figures**

**Supplemental Figure 1. Study design and enrollment of patients.** Four institutions participated in this research. The patients who met the inclusion or exclusion criteria were enrolled in the current analysis. The primary endpoint was RFS, and the secondary endpoint was OS.

**Supplemental Figure 2. RFS, OS, and PRS curves of all patients according to each individual risk factor.** (A) RFS, (B) OS, (C) PRS. The proportional risk of each factor was confirmed by curve shape.
