# Supplementary figures and images for: Long-term follow-up of a consecutive cohort validating an epidermal growth factor receptor mutation as an independent risk factor for postoperative recurrence in lung adenocarcinoma
Source: Interdiscip Cardiovasc Thorac Surg. 2023 Oct 31;37(5):ivad174. doi: 10.1093/icvts/ivad174 (PMC10640389; doi:10.1093/icvts/ivad174)

## (A) Recurrence-free survival

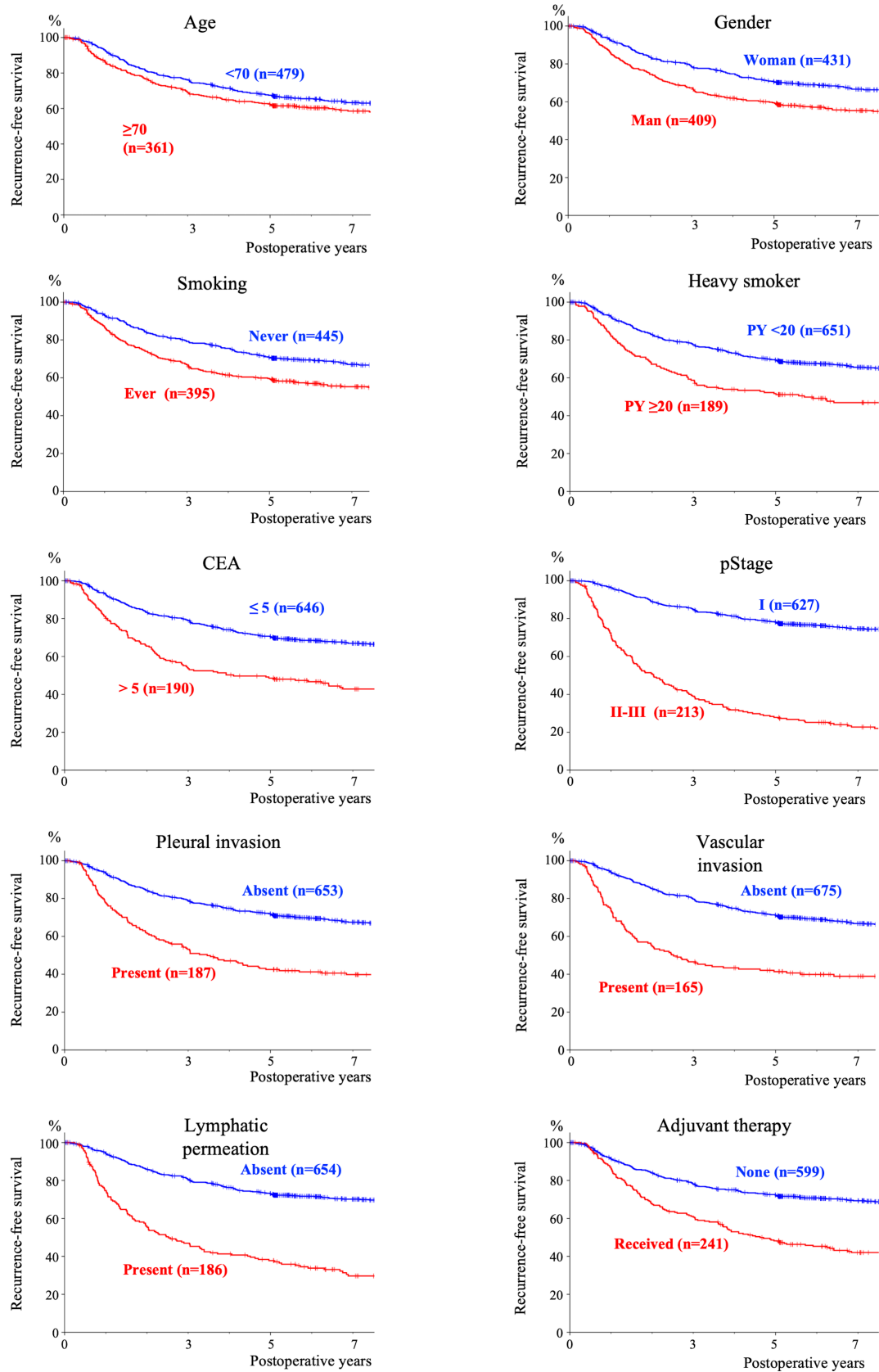

Supplement: ivad174_Supplementary_Data [file ivad174_supplementary_data.zip › Suppl fig2A.pdf]

## (B) Overall survival

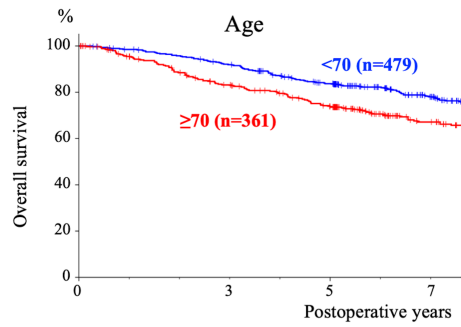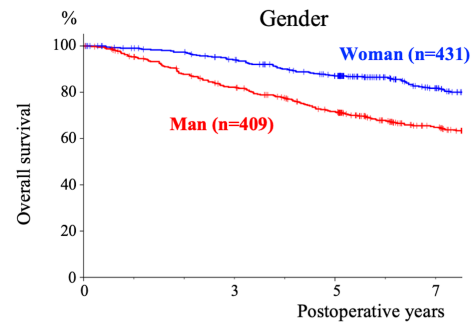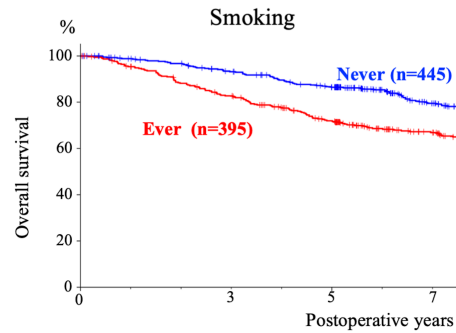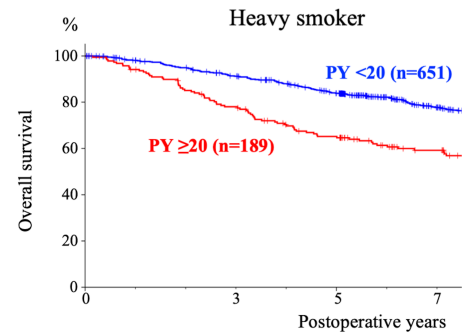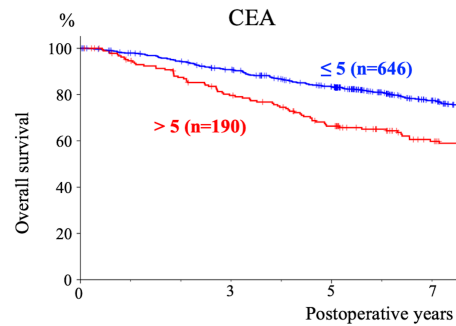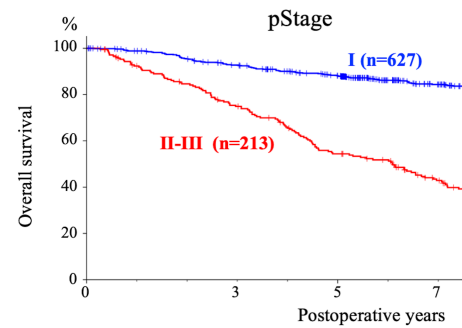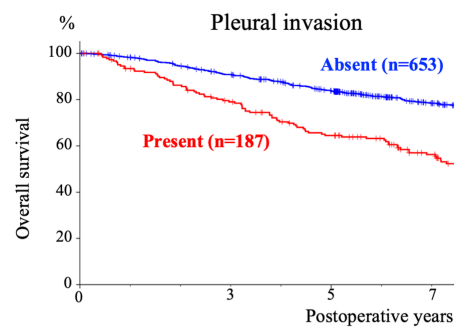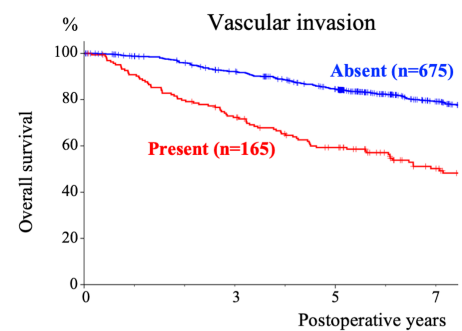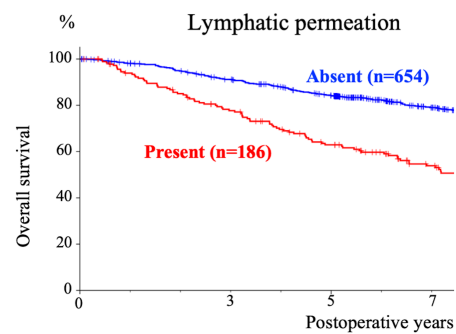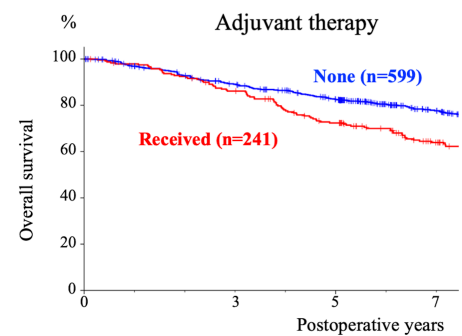

Supplement: ivad174_Supplementary_Data [file ivad174_supplementary_data.zip › Suppl fig2B.pdf]

### (C) Postrecurrence survival

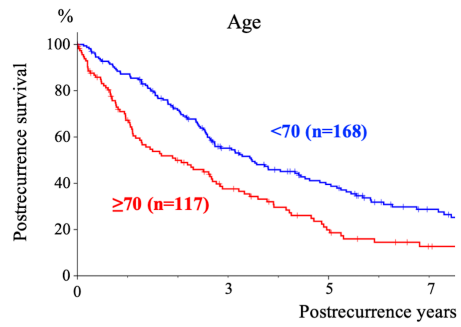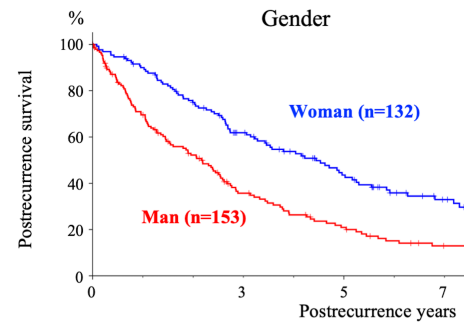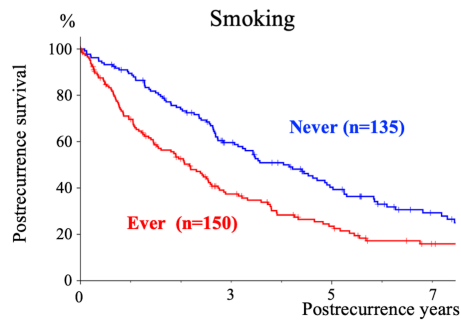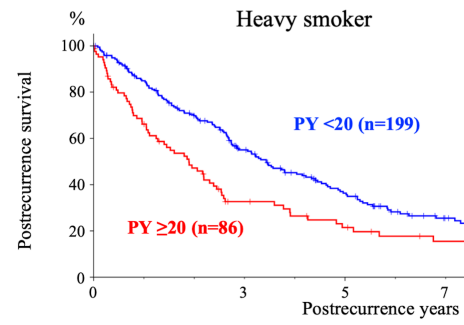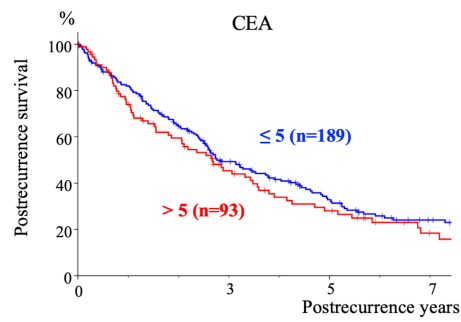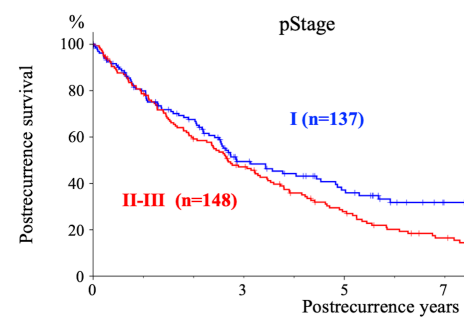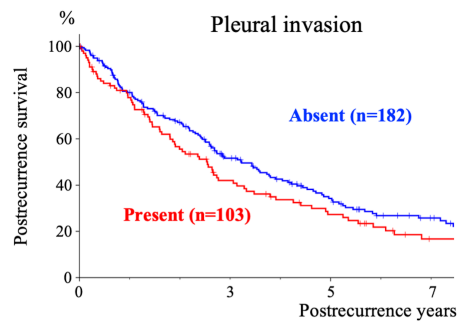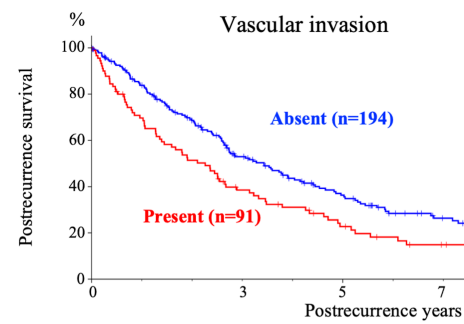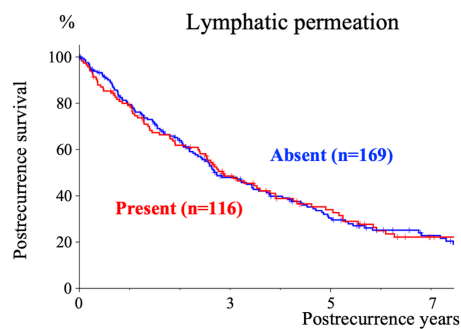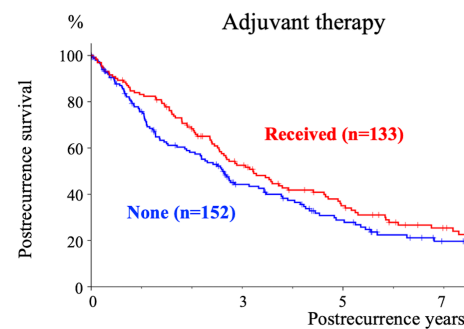

Supplement: ivad174_Supplementary_Data [file ivad174_supplementary_data.zip › Suppl fig2C.pdf]
